# Skeletal interoception in osteoarthritis

**DOI:** 10.1038/s41413-024-00328-6

**Published:** 2024-04-01

**Authors:** Dinglong Yang, Jiawen Xu, Ke Xu, Peng Xu

**Affiliations:** 1 Department of Joint Surgery, Honghui Hospital, Xi’an Jiaotong University, Xi’an, 710054 China; 2 Department of Orthopedic Surgery and Orthopedic Research Institute, West China Hospital, Sichuan University, Chengdu, 610041 China

**Keywords:** Bone, Pathogenesis

## Abstract

The interoception maintains proper physiological conditions and metabolic homeostasis by releasing regulatory signals after perceving changes in the internal state of the organism. Among its various forms, skeletal interoception specifically regulates the metabolic homeostasis of bones. Osteoarthritis (OA) is a complex joint disorder involving cartilage, subchondral bone, and synovium. The subchondral bone undergoes continuous remodeling to adapt to dynamic joint loads. Recent findings highlight that skeletal interoception mediated by aberrant mechanical loads contributes to pathological remodeling of the subchondral bone, resulting in subchondral bone sclerosis in OA. The skeletal interoception is also a potential mechanism for chronic synovial inflammation in OA. In this review, we offer a general overview of interoception, specifically skeletal interoception, subchondral bone microenviroment and the aberrant subchondral remedeling. We also discuss the role of skeletal interoception in abnormal subchondral bone remodeling and synovial inflammation in OA, as well as the potential prospects and challenges in exploring novel OA therapies that target skeletal interoception.

## Introduction

Interoception is the fundamental basis of human perception and cognition, encompassing the intricate processes of perceiving, integrating, and interpreting signals originating from organs. Through interoception, the brain effectively assesses and regulates the physiological status of internal organs, such as blood pressure and visceral pain.^1^ Conversely, exteroception pertains to the perception of diverse sensations derived from the external environment, such as vision, touch, and temperature.^2^ Humans achieve adaptive behavior by integrating interoceptive and exteroceptive information derived from their internal body systems and the surrounding external world.^3^ The skeleton, one of the largest organs, is composed of intricately connected bones that provide a stable internal environment for mineral storage and functional hematopoiesis.^4^ Sensory and autonomic nerves are widely distributed across the surfaces of bones and joints, intricately regulating the physiological function and metabolism of the skeletal system through complex interoceptive mechanisms. The interoceptive mechanisms related to bone metabolism and homestasis are called skeletal interoception.^5^

Osteoarthritis (OA) is a significant component of skeletal diseases. With the global population aging, OA affects more than 500 million population worldwide.^6^ OA is characterized by progressive cartilage degeneration and pathological alterations in the subchondral bone and surrounding soft tissues, leading to long-term chronic pain.^7^ The pathogenesis of OA involves mechanical, inflammatory, and metabolic factors that interact with each other. However, previous research has primarily focused on exploring local molecular regulation, epigenetics, and metabolomics mechanisms in OA. The limited understanding of OA results in suboptimal treatment outcomes. Advanced OA can only be effectively treated with joint replacement surgery.^8,9^ Since the organism functions as a cohesive unit, it is plausible to consider whether the brain modulates the physiological functions of joints, implying a potential correlation between skeletal interoception and the pathogenesis of OA.

The skeletal interoceptive circuit consists of sensory nerve - brain - sympathetic nervous system (SNS).^5^ Previous study found that mechanical load leads to increased levels of prostaglandin E2 (PGE2) in bone. The elevated levels of PGE2 transmit interoceptive signals through sensory neurons to the hypothalamus, where the interoceptive signals are integrated and interpreted. Subsequently, the hypothalamus regulates bone homeostasis via the descending sympathetic pathway.^10^ The subchondral bone sclerosis, and bone cyst and osteophyte formation are pathological features of advanced OA,^11^ which are associated with alterations in bone matrix biochemistry and mechanical properties.^12^ It is clear that the subchondral bone of knee and hip joints bears the load of gravity, which is likely to be perceived by the brain through skeletal interoception. Excessive mechanical loads induce the increase of PGE2 in subchondral bone, which contributes to the progression and pain of OA through sensory nerves.^13,14^ In animal models, targeting skeletal interoception has shown significant effects in inhibiting OA progression and providing sustained relief from OA pain.^13,15,16^ Uncovering the mechanisms of skeletal interoception may provides a promising avenue for the development of conservative therapies and pain management for OA.

In this comprehensive review, we elucidated the conception and components of interoception, with a specific focus on skeletal interoception. We discussed the role of skeletal interoception in mediating abnormal subchondral bone remodeling and synovial inflammation in OA. Furthermore, we explored the research progress in targeting skeletal interoception for the treatment of OA and its pain. The aim of this review is to underscore the critical role of skeletal interoception in the pathogenesis of skeletal disorders, particularly OA, and provide research directions and insights for the treatment of OA.

## The conception of interoception

**Fig. 1:**
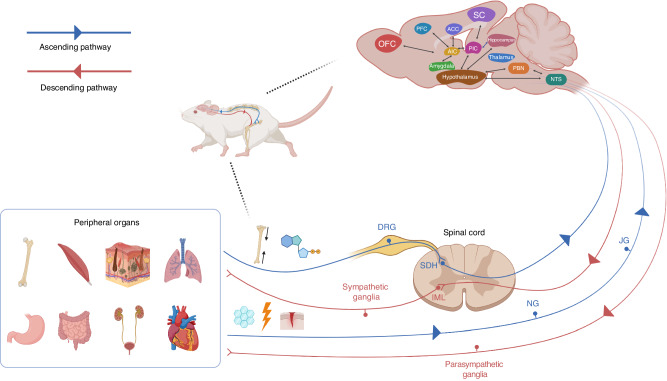
Diagram of the interoception circuits. The interoceptors located at the peripheral sensory nerve terminals perceive interoceptive signals generated by peripheral organs. The spinal and vagal nerves are two major ascending/afferent pathways that transmit interoceptive signals to the brain. In spinal nerve pathway, the somatosensory neurons in DRG project to the brain via the SDH. The spinal nerves primarily transmit somatic sensations such as temperature, injury, and pain-related signals from the skin, muscle, and skeleton. The vagal nerves transmit mechanical and chemical signals originating from visceral organs through JG and NG. Upon receiving interoceptive signals, various barin regions, including the NTS, thalamus, hypothalamus, PBN, hippocampus, ACC, amygdala, SC, PIC, AIC, PFC, and OFC, partake in the integration and interpretation of these signals. Finally, the brain sends the regulatory signals back the origin organs of interoceptive signals through descending/efferent interoceptive pathways. The SG, receiving projection from preganglionic sympathetic neurons in the spinal IML, and the PSG are two interoceptive effectors to maintain the internal organ homeostasis. This figure is a modified version of Fig. 1 published in *Cell Metab Dec 6;34(12):1914-1931*. DRG dorsal root ganglia, SDH spinal dorsal horn, JG jugular ganglia, NG nodose ganglia, NTS nucleus tractus solitarius, PBN parabrachial nucleus, ACC anterior cingulate cortex, SC somatosensory cortex, PIC posterior insular cortex, AIC anterior insular cortex, PFC prefrontal cortex, OFC orbitofrontal cortex, SG sympathetic ganglia, IML intermediolateral nucleus, PSG parasympathetic ganglia

In 1906, Sherrington first proposed the concept of interoception, which is defined as the intricate interplay between the brain and peripheral organs to represent and monitor an organism’s internal state.^17^ Over the years, this definition has undergone evolution to encompass a more dynamic portrayal of the organism’s physiology. This includes the intricate process by which the central nervous system (CNS) senses, integrates, and regulates signals pertaining to the internal state of the body to maintain homeostasis.^18^ Interoceptors receive interoceptive signals from peripheral organs and transmit them to the brain via ascending pathways. Specific regions within the brain, such as the hypothalamus and insula, integrate and interpret interoceptive signals and subsequently release regulatory signals through descending pathways to maintain internal homeostasis (Fig. 1).^1,19^ Interoceptive signals are highly precise, meaning that the signals always return to their origin.

In this section, we elucidate the ascending and descending interoceptive pathways. We also discuss how the brain receives and processes afferent interoceptive signals, and maintain internal homeostasis through descending pathways.

### Ascending interoceptive pathways

Various interocetpors, including chemoreceptors, hormone receptors, and mechanoreceptors, are distributed in different tissues and organs.^20^ These receptors detect dynamic changes in the internal environment and generate interoceptive signals. Two major ascending pathways transmit interoceptive signals to brain. In the first ascending pathway, the somatosensory neurons in dorsal root ganglia (DRG) project to the brain via the spinal dorsal horn (SDH). This pathway primarily conveys somatic sensations such as temperature, injury, and pain-related signals from the skin, muscle, and skeleton.^21,22^

In the second ascending pathway, interoceptive signals are transmitted through sensory ganglia, such as jugular and nodose ganglia in the tenth cranial nerve, that sends axons to the brain stem.^23^ The vagal ascending pathway is the primary conduit for transmitting mechanical and chemical signals originating from visceral organs, spanning from the rostral to caudal.^24,25^ Sensory and motor vagal nerve fibers transmit visceral signals to the brain and exert physiological responses, which orchestrates the whole-body metabolic homeostasis.^26^

### The signal interpretation in the brain

Interpreting interoceptive signals within the brain involves intricate interactions between various brain regions. Interoceptive information is firsty transmitted to subcortical region, such as nucleus tractus solitarius (NTS), which serves as a classic area for visceral reception.^27^ Subsequently, the interoceptive information is conveyed to key regions including the thalamus, hypothalamus, parabrachial nucleus (PBN), and hippocampus. The hypothalamus and PBN mainly process information related to metabolism, pain, temperature, and olfaction.^28–30^ Finally, the higher-level brain regions, including the insula, anterior cingulate cortex (ACC), amygdala (AMY), primary and secondary somatosensory cortex (SC), as well as orbitofrontal cortex (OFC) and medial prefrontal cortex (PFC), may receive and further interpret the interoceptive signals.^23,31–39^ The insular cortex, comprising the posterior insular cortex (PIC) and the anterior insular cortex (AIC), is a relatively underestimated brain area in neuroscience.^40^ Initially, the primary interoceptive signals reach the PIC where low-level sensory features undergo processing. Subsequently, this refined information is relayed to the AIC, which plays a pivotal role in nurturing subjective feeling states.^41^ However, no neuron is an island. The AIC exhibits bidirectional connectivity with the ACC, PFC, OFC, and AMY, forming a comprehensive functional network.^19,42^

The functionality of the brain is incredibly vast and complex. It not only processes various sensory signals and regulates disease states but also influences an individual’s psychological state and emotional responses. Despite centuries of research on brain function, our understanding of the brain remains a grain of sand in a vast desert.

### Descending interoceptive pathways

After receiving interoceptive signals, the brain fully interprets them and releases regulatory signals to target organs. The neurons involved in generating these regulatory signals within the brain generally are regarded as the central regulatory factors of interoception.^19^ The main role of descending signals of interoception is to maintain the homestasis of internal organs.

The efferent interoceptive signals are transmitted through autonomic nervous system (ANS), which consists of two branches: sympathetic and parasympathetic nervous systems (PSNS).^43–45^ In SNS, the descending interoceptive pathway derived from the hypothalamus may reach the preganglionic neurons of the sympathetic ganglia, which are located in the spinal intermediolateral nucleus (IML), spanning from the first thoracic to the second lumbar segments.^46^ The projections from preganglionic sympathetic neurons are received by the paravertebral and prevertebral sympathetic ganglia or chromaffin cells in the adrenal medulla. The SNS maintains the homeostasis of peripheral organs, primarily through the neurotransmitters namely adrenaline and norepinephrine (NE). Adrenaline and NE are catecholamines that elicit physiological responses such as increasing heart rate and blood pressure and inhibiting digestive function. These effects are mediated by their combination with the adrenergic receptors (AR). Alternatively, the efferent interoceptive signals are transmitted through parasympathetic neurons originating from the cranial nerves. The PSNS predominantly utilizes acetylcholine for neurotransmission,^47,48^ which interacts with acetylcholine receptors to produce effects opposite to those of adrenaline and NE. By coordinating the effects of different signaling molecules such as adrenaline, NE, and acetylcholine, the organism can fine-tune its responses and maintain a delicate equilibrium.

### Skeletal interoception

The skeletal system functions as a supportive framework for the whole body and is the major regulator of hematopoietic function.^49^ The skeleton also functions as an endocrine organ, regulating the global homeostasis of minerals, fats, and glucose.^50^ Bone is a dynamic organ under constant remodeling to adjust its density and architecture.^51^ Bone remodeling mainly occurs in the bone marrow and is accomplished by osteoclasts (OCs) mediated bone resorption and osteoblasts (OBs) mediated bone formation. OCs serve as the “conductors” of the “symphony” of bone remodeling.^52^ The magnitude and direction of mechanical loads are determinant factor of bone remodeling. The bone is extensively innervated by sensory and sympathetic nerves,^46,53^ and it is well recognized that the CNS plays a crucial role in regulating bone homeostasis.^54^ However, how the brain perceives changes in mechanical load and density of bone and regulates bone homeostasis through the SNS remains unknown. As mentioned in “introduction”, skeletal interoception is mainly initiated by PGE2, consequently, skeletal interoception is also called “PGE2 interoception”.^5^ Owning to the limited space, please reference the review by Lv et al. for more comprehensive information regarding skeletal interoception.^5^

## Subchondral bone microenvironment and subchondral bone remodeling in OA

In this section, we provide an overview of the normal subchondral bone microenvironment. Furthermore, we delve into the etiology, histopathological alterations, and biology mechanisms of abnormal subchondral bone remodeling. Finally, we discuss the mechanisms underlying excessive innervation of sensory nerves in the OA subchondral bone. This section aims at laying the groundwork for readers to better understand the role of skeletal interoception in OA progression and pain.

### Subchondral bone microenvironment

Subchondral bone refers to the subchondral bone plate and subchondral trabeculae beneath the articular cartilage. The subchondral bone plate located just beneath the calcified cartilage, is a a thin cortical plate.^55^ Adjacent to the subchondral bone plate, the subchondral trabeculae represent porous structures abundant in vessels and nerves. These structures not only provide essential load absorption and structural support but also serve as crucial suppliers of nutrients to the cartilage.^56^ The mesenchymal stem cell (MSC)-derived OBs and monocyte/macrophage (Mo/Mac)-derived OCs coordinate to remodel subchonral bone. The subchondral bone undergoes continuous remodeling to adapt to dynamic mechanical forces exerted on the joint and replace damaged bone with new bone.^57,58^ However, the uncoupling bone remodeling contributes to the histopathological alterations in the OA subchondral bone microenvironment and subsequent cartilage erosion.^59^

### Uncoupling of bone remodeling in the OA subchondral bone microenvironment

**Fig. 2:**
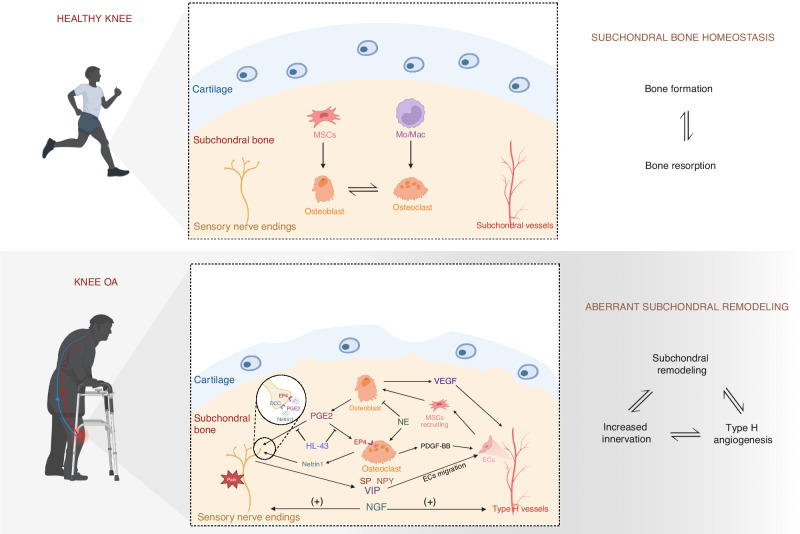
Aberrant subchondral bone remodeling in OA. OB-derived PGE2 interacts with EP4 receptors on OCs, promoting bone resorption and inducing the secretion of PDGF-BB to enhance type H angiogenesis. Furthermore, PGE2 stimulates OCs to secrete netrin1, which interacts with the DCC receptor on CGRP^+^ sensory nerves, leading to increased innervation in subchondral bone. In the OA subchondral bone, CGRP^+^ nerves stimulate EC migration through the release of SP, NPY, and VIP, which promote type H angiogenesis. The ECs involved in angiogenesis recruit MSCs through chemotaxis, thereby facilitating bone formation. The VEGF derived from OBs stimulates angiogenesis. The NGF stimulates both vascular and neural growth. Generally, the activation of SNS leads to subchondral bone resorption while suppressing bone formation through NE. OB osteoblast, PGE2 prostaglandin E2, EP prostaglandin E, OC osteoclast, PDGF-BB platelet-derived BB growth factor, DCC deleted in colorectal cancer, CGRP calcitonin-gene-related peptide, EC endothelial cell, SP substance P, NPY neuropeptide Y, VIP vasoactive intestinal peptide, MSC mesenchymal stem cells, VEGF vascular endothelial growth factor, NGF nerve growth factor, SNS sympathetic nervous system, NE norepinephrine

Abnormal subchondral bone remodeling is linked to alterations in bone matrix biochemistry (Table 1) and mechanical properties, which significantly contribute to the pathophysiology of OA.^12^ In response to mechanical loading or other uncertain etiology, alterations occur in the turnover rate of subchondral bone. In early OA, the subchondral bone turnover rate increases, leading to the subchondral bone plate thinner and more porous. The subchondral trabeculae deteriorate, characterized by heightened trabecular spacing and diminished trabecular thickness. In late OA, the subchondral bone plate and trabeculae thicken, leading to subchondral bone sclerosis and decreased trabeculae separation, which is accompanied by aberrant excessive type H angiogenesis and sensory nerve innervation (Fig. 2).^60,61^ Increased OB differentiation mediated by skeletal interoception may be one of the mechanisms underlying subchondral bone sclerosis in advanced OA. More information will be disscused in following sections. Despite the increased bone mass, the diminished calcium content, inadequate mineralization, and reduced bone elasticity, compromise the capacity to withstand mechanical loads.^62^ The aberrant subchondral bone architecture promotes chondrocyte apoptosis and cartilage degradation.^63^ Anomalous cartilage exerts excessive pressure on the subchondral region, consequently intensifying the turnover rate triggered by mechanical forces and establishes a positive feedback loop that accelerates the progression of OA.

**Table 1:** The role of main biochemistry substances in OA

Names	Receptors	Roles	References
Substance P (SP)	Neurokinin 1 receptor (NK1R)	Promote angiogenesis in the subchondral bone; contribute to cartilage degradation and synovial inflammation; mediate joint pain	^158–162^
Prostaglandin E2 (PGE2)	Prostaglandin E (EP) 1–4 receptors	Disturb subchondral bone remodeling; promote innervation in the subchondral bone; regulate chondrocytes metabolism; mediate joint pain	^10,14,15,163^
Nerve growth factor (NGF)	Tropomyosin receptor kinase A (TrkA)	Promote vascular and nerve growth in the subchondral bone; mediate hyperalgesia of OA synovium; mediate mechanical load-induced pain	^164–166^
Transforming growth factor (TGF)-β	TGF-β receptor 1–3	Contribute to cartilage degeneration; lead to aberrant bone formation accompanied by increased angiogenesis	^61,167^
Calcitonin-gene-related peptide (CGRP)	Calcitonin receptor-like receptor (CRLR)/ receptor activity modifying protein (RAMP) 1	Promote apoptosis and senescence of chondrocytes; inhibit the expression of chondrogenic markers	^168^
α-melanocyte-stimulating hormone (α-MSH)	Melanocorticosteroid receptor 1 (MC1R)	Inhibit inflammation in the synovium and cartilage; play a chondroprotective role in cartilage	^169,170^
Platelet-derived BB growth factor (PDGF-BB)	Platelet-derived growth factor receptor (PDGFR)-α; PDGFR-β	Induce abnormal angiogenesis and bone remodeling in OA subchondral bone	^14,171,172^
Norepinephrine (NE) or noradrenaline (NA)	α1, α2, β1, β2, β3-adrenergic receptor (AR)	Protect cartilage from degeneration (β-AR); inhibit subchondral bone formation (β2-AR); induce the apoptosis of chondrocytes (α1-AR); inhibit the chondrogenic differentiation of synovial adipose stem cell (α2- AR)	^96,173–176^
Vasoactive intestinal peptide (VIP)	Vasoactive intestinal peptide receptor (VIPR) 1, 2	Inhibit cartilage extracellular matrix (ECM) degradation; alleviates OA via inhibiting NF-κB signaling pathway	^177–179^
Neuropeptide Y (NPY)	Y receptor 1 (Y1R); Y2R; Y4R; Y5R	Increases in OA synovial fluid and correlate to Watanabe pain scores; increases in dorsal root ganglion (DRG) of OA; exacerbate the OA progression	^180–182^
Serotonin (5-HT)	5-HT 1-7 receptors	Repress cartilage regeneration; promote chondrocyte inflammation	^183,184^

### Increased innervation of sensory nerves in OA subchondral bone microenvironment

Pain is the primary symptom in patients with OA and the reason driving patients to seek treatment. The pain mechanisms of OA were further elucidated until researches uncovered the increased innervation of sensory nerves that respond to noxious stimuli in OA subchondral bone.^64^ The innervation of sensory nerves in the subchondral bone is linked to complex cellular interactions and neuro-related biochemical factors. Nerve growth factor (NGF), a pleiotropic molecule, guides nerve sprouting and innervation in bone. Mechanical load stimulates the OB proliferation accompanied by increased NGF secreation in subchondral bone.^65^ During the process of bone remodeling, OC-mediated bone resorption creates space for nerve sprouting and promotes the innervation of nerves through the release of nerve inducing molecules, such as netrin 1 and NGF. The receptor activator of nuclear factor κB ligand (RANKL) on OBs can bind to RANK on OC precursors. The activated OC precursors releasing NGF, are key drivers of nerve innervation in the subchondral bone of OA.^66^ OCs are the primary source of netrin 1 in subchondral bone, which promotes nerve sprouting by binding to the deleted in colorectal cancer (DCC) receptors on calcitonin gene-related peptide (CGRP)^+^ sensory nerves.^14^ The leptin receptor-expressing (LepR)^+^ stromal cells also serve as crucial sources of NGF in bone marrow,^67^ indicating that the LepR^+^ stem cells may play a pivotal role in nerve innervation in OA subchondral bone marrow. The acidic microenvironment resulting from bone resorption and the inflammatory microenvironment in OA subchondral bone contributes to the hyperalgesia of nociceptive neurons.^68,69^ Consequently, increased sensory nerve innervation links nociceptive and hypersensitive pain in OA. Targeting aberrant innervation of sensory nerves is a promising therapy for OA pain.

## Abnormal mechanical loading disturbs subchondral bone homeostasis through skeletal interoception

### Excessive mechanical load stimulates PGE2 production in the subchondral bone

According to Wolff’s law, the internal architecture of bone is determined by the magnitude and direction of the applied load.^70^ The subchondral bone requires continuous adaptation to mechanical stimuli through bone remodeling.^71^ Excessive mechanical loads promotes subchondral bone formation and sclerosis, and OA progression, which is one of the reasons why obesity exacerbates OA.^72,73^ Anterior cruciate ligament transection (ACLT) and destabilization of the medial meniscus (DMM), the most commonly used methods to induce OA animal models, contribute to abnormal joint load, and the subsequent subchondral bone and cartilage lesions.^74,75^ In some patients with OA, the mechanical load on subchondral bone increases due to factors like improper joint alignment, thinning of cartilage, and decreased ability of subchondral bone to absorb load.^62,76,77^ The CNS maintains organ homeostasis by perceiving and integrating various stimuli. Mechanical stimulation serves as a crucial interoceptive signal for maintaining skeletal homeostasis.^78^ However, how mechanical interoceptive signals within the joint are perceived by the brain remains a subject of inquiry.

**Fig. 3:**
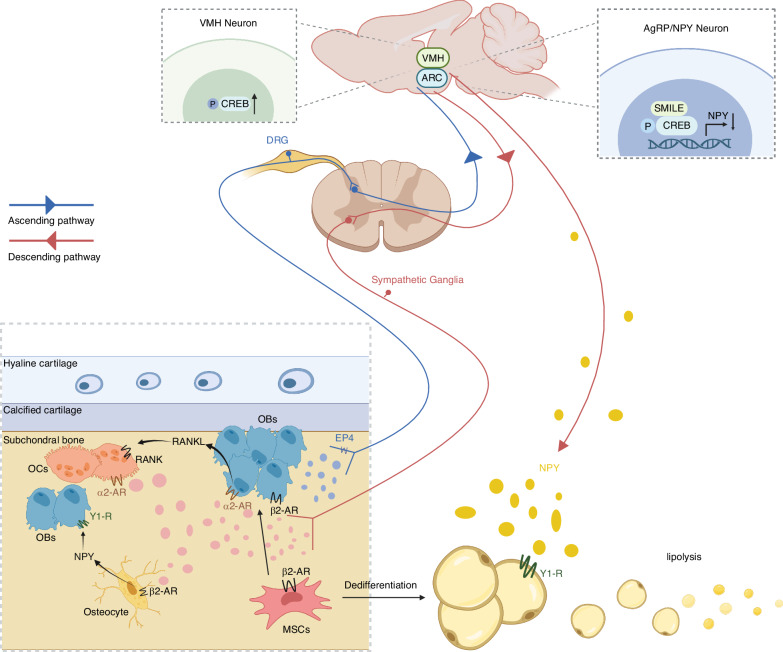
Skeletal interoception in the aberrant subchondral bone remodeling of OA. Mechanical load stimulates OB-derived PGE2 production in the subchondral bone. The elevated PGE2 levels acting on EP4 receptors of sensory nerves transmits interoceptive signals to the VMH, leading to the activation of CREB signaling pathway. The activation of CREB signaling downregulated the descending sympathetic tone, leading to the commitment of MSCs to an osteoblastic lineage and promoting osteogenesis. The downregulated SNS promotes OB proliferation and differentiation via β2-AR, suppresses OC proliferation via α2-AR, and inhibits bone resorption through the α2-AR on OBs and decreased RANKL expression. Moreover, decreased NE binding with β2-AR on osteocytes inhibits NPY production, the decreased NPY locally promotes OB proliferation as well. The skeletal interoceptive signaling induces the expression of hypothalamic SMILE. The SMILE forms a transcriptional heterodimer with phosphorylated CREB on *Npy* promoters to suppress NPY expression. The downregulated hypothalamic NPY stimulates the lipolysis of adipose tissue to supply energy for osteoblastic bone formation. OB osteoblast, PGE2 prostaglandin E2, EP prostaglandin E, VMH ventromedial nucleus of hypothalamus, cAMP cyclic adenosine monophosphate, CREB cAMP response element-binding protein, MSC mesenchymal stem cell, SNS sympathetic nervous system, AR adrenergic receptor, RANKL receptor activator of nuclear factor κB ligand, NE norepinephrine, NPY neuropeptide Y, SMILE small heterodimer partner-interacting leucine zipper protein

Previous research has identified PGE2 as a key regulator of bone formation.^79^ Consequently, researchers explored whether PGE2 levels are associated with mechanical loading and promote bone formation through skeletal interoceptive signaling. The mechanical loads stimulate the secretion of OB-derived PGE2 in femurs accompanied by increased bone mass.^10^ To simulate microgravity conditions, Guo and colleagues used hindlimb unloading (HU) mice models and observed a notable reduction in PGE2 content in unloading bones. Correspondingly, bone mass in unloading bones also significantly decreased.^80^ The osteocytes and OBs have been identified as mechanosensitive cells in subchondral bone.^81,82^ In the DMM and ACLT mouse models of OA, there is a substantial elevation in PGE2 levels in the subchondral bone.^13–15^ Similarly, PGE2 is highly secreted by OBs in the subchondral bone of OA patients.^14^ The increased presence of PGE2 contributes to the abnormal osteogenesis in the OA subchondral bone (Fig. 3). Intriguingly, the deletion of OB prostaglandin E (EP) 4 receptors does not alter bone density in mice, suggesting that PGE2-induced bone formation is mediated through alternative pathways from cell autonomous EP4 signaling, such as skeletal interoception pathway.^83^ However, how the brain perceives the changes in PGE2 levels?

### PGE2 transmits interoceptive signals to brain via EP4 receptors on sensory nerves

The PGE2 level in the subchondral bone is elevated in both patients and mice with OA. The subchondral bone is richly innervated by sensory nerves that interact with the CNS.^59^ However, it remains unclear whether the sensory nerves in the subchondral bone can perceive changes in PGE2 levels and how they transmit PGE2 interoceptive signals to the brain.

Among the four receptors (EP1-4) of PGE2, EP4 serves as the primary receptor for bone formation.^84^ It was found that EP4 receptors are expressed in sensory nerves of bone by using coimmunofluorescent staining of EP4 with CGRP. To validate the function of EP4 in sensory nerves, *EP4*_Avil_^−/−^ mice were used to induce the ablation of EP4 in sensory nerves by crossing Advillin-cre mice with *EP4*^wt^ mice. The results showed a significant decrease in both cortical and trabecular bone in the EP4-ablated mice at 12 weeks.^10^ In DMM OA mice, EP4 exhibited a notable increase in the entire joint. Sensory denervation or knockout of EP4 receptors on sensory nerves effectively suppressed abnormal subchondral bone remodeling and reduced OA severity.^13^ In summary, sensory nerves in the subchondral bone detect changes in PGE2 level and affect subchondral bone remodeling through the EP4 signaling.

Advancements in cellular techniques, such as immunofluorescence and viral tracing experiments, have well elucidated the route from the skeletal sensory neurons to the CNS.^21,85^ The DRG contains the cell bodies of sensory neurons innervating the knee joint.^86^ The skeletal sensory neurons in L1–L6 DRG converge with second order neurons in the SDH, projecting to the brain.^87^ Today, it is well-established that the hypothalamus serves as a hub for receiving, analyzing, and harmonizing sensory inputs, thereby facilitating a bidirectional communication with various behavioral, autonomic, and endocrine pathways. The various individual nuclei of hypothalamus is associated with feeding behavior, energy balance, thermoregulation, stress responses, fluids and electrolyte homeostasis, reproduction, circadian rhythms, and bone homeostasis.^88,89^ Due to the association between bone mass and the cAMP (cyclic adenosine monophosphate) response element-binding protein (CREB) signaling pathway in the ventromedial nucleus of hypothalamus (VMH),^90^ Chen and colleagues explored the role of CREB signaling in skeletal interoception, and found that PGE2/EP4 axis transmits signals to VMH, leading to the activation of CREB signaling pathway to regulate bone formation.^10^ The neurons coexpressing orexigenic neuropeptide Y (NPY) and AgRP (NPY/AgRP) in hypothalamus arcuate nucleus (ARC) play a crucial role in foraging and regulating lipid metabolism to meet the energetic requirements of OB-driven bone formation.^91^ Unloading-induced skeletal interoception stimulates the expression of NPY in ARC to supply energy for bone formation in HU model.^80^ Collectively, PGE2 binds to the EP4 receptors on sensory nerves in the subchondral bone, transmitting skeletal interoceptive signals to the hypothalamus through the DRG, thereby affectting subchondral bone remodeling. It is worth exploring in the future whether other hypothalamic nuclei, such as the paraventricular (PVN), dorsomedial (DMH), hypothalamic area (LH), anterior hypothalamus (AH), and posterior hypothalamic (PH) nuclei, which are associated with energy metabolism and stress response, are also involved in skeletal interoception.

### Downregulated sympathetic activity promotes subchondral bone formation

It has been defined that hypothalamic-ANS serve as efferent neuronal pathway regulating skeletal metabolism.^92^ Generally, the activation of the SNS leads to bone resorption while suppressing bone formation, whereas the PNS exerts contrasting effects.^93^ Mechanistically, the SNS releases NE, which bind to α1, α2, β1, β2, β3-AR to exerts its physiological effects. The binding of NE with β2-AR on OBs inhibits their proliferation and differentiation, while its binding with α2-AR on OBs promotes their RANKL expression, thereby mediating bone resorption.^94^ Moreover, NE binding with β2-AR on osteocytes stimulates NPY production, which locally inhibits OB proliferation as well.^95^ In healthy bone, the innevating sympathetic and parasympathetic nerves orchestrate to maintain subchondral bone homeostasis. Pevious section has elucidated that in subchondral bone of advanced OA, the excessive OB-mediated bone formation results in subchondral bone sclerosis, which is induced by the imbalanced activity of SNS and PNS. For example, the OA mice with deficiency in β2-AR (*Adrb2*^–/–^) exhibit enhanced OB activation and increased subchondral bone plate thickness.^96^ More intuitively, sympathectomy generated by 6-hydroxydopamine aggravates OA-specific cartilage calcification and subchondral bone thickening.^97^ For a more comprehensive understanding of the ANS in OA subchondral bone remodeling, please refer to a previous review.^98^

Considering the pivotal role of the SNS in subchondral bone remodeling, it is worth exploring whether the SNS serves as the descending pathway for skeletal interoception. Previous study found that the activation of CREB signaling in VMH by the OB-derived PGE2/EP4 skeletal interception downregulates sympathetic nerve activity. The downregulated sympathetic tone leads to the commitment of MSCs to an osteoblastic lineage and promoting osteogenesis.^99^ Increased sympathetic tone in sensory denervation models contributes to bone loss, but administration of a β2-AR antagonist rescues this effect.^10^ Conversely, in HU model, reduced PGE2 interception signal stimulates the expression of hypothalamic tyrosine hydroxylase (TH), a marker of sympathetic activity, resulting in bone loss.^80^

The NPY expressed in the central and peripheral nerves regulates the whole-body energy metabolism through the CNS.^100^ A significant expenditure of energy is essential for bone formation, the reduced hypothalamic NPY stimulates the lipolysis of adipose tissue to supply energy for osteoblastic bone formation. The skeletal interoceptive signaling induces the expression of hypothalamic small heterodimer partner-interacting leucine zipper protein (SMILE). The SMILE forms a transcriptional heterodimer with phosphorylated CREB on *Npy* promoters to suppress NPY expression, which is increased in the hypothalamus of HU model.^80,91^ In summary, the SNS acts as the efferent pathway of skeletal interoception for regulating subchondral bone remodeling. The hypothalamic NPY is also modulated by skeletal interoception to provide energy for osteoblastic bone formation. Further exploration is warranted to determine whether the PNS is also implicated in the skeletal interoception as a descending pathway.

## The NGF-TrkA Signaling Activated by Mechanical Loads is a Potential Mechanism of Skeletal Interoception

The NGF is a highly conserved polypeptide belonging to the small family of neurotrophins. It binds selectively to the tropomyosin receptor kinase A (TrkA) receptor, initiating various signaling pathways associated with neural cell survival, proliferation, and differentiation.^101^ Approximately 80% of the nerves distributed on the surface of adult bones express TrkA, a sensory nerve receptor.^102,103^ Previous studies have indicated that TrkA sensory nerves in mice innervating the developing femur contribute to the formation of primary and secondary ossification centers.^87^ In order to delve deeper into the role of TrkA and its ligand NGF in mechanically adaptive bone formation, Tomlinson et al. uncovered a bidirectional communication between OBs and sensory nerves in mouse bone.^65^ Their research revealed that in vivo mechanical loading induces a significant upregulation of NGF expression in OBs within one hour of loading, and administration of exogenous NGF to wild-type mice was found to significantly increase load-induced bone formation. Conversely, the knockout of TrkA signaling in mice harboring mutant TrkA alleles greatly attenuated axial compression-induced bone formation.^65^ A Subsequent investigation further demonstrated the importance of TrkA signaling in stress fracture repair, suggesting that skeletal TrkA sensory nerves serve as crucial upstream mediators in this reparative process.^104^ In summary, alongside the PGE2/EP4 signaling, NGF-TrkA represents another potential skeletal interoceptive signal contributing to the OA subchondral bone formation.

While this research provides exciting new clues regarding skeletal interoception, further investigations are required to validate NGF-TrkA as an additional potential interoceptive signal governing the OA subchondral bone formation: (a) It remains to be explored whether mechanical loading elicits a similar upregulation of OB-derived NGF in the subchondral bone, consequently driving bone formation through the sensory nerve TrkA signaling; (b) The NGF-TrkA signaling influences the signaling pathways or expression of neurogenic substances in the hypothalamic nuclei; (c) The SNS or PNS serve as descending pathway of NGF-TrkA signaling affecting subchondral bone formation.

## The potential role of skeletal interoception in OA synovitis

### The synovitis is one of the OA characteristic

The synovium, which is located in the innermost layer of the joint cavity, participates in lubrication, nutrition, and removal of metabolic byproducts from the joint cavity.^105,106^ Normal synovial tissue (ST) is composed of fibroblasts and macrophages.^107^ They perform specific functions within the articular system, including secreting lubricin, host defense, and immune regulation, thereby maintaining joint homeostasis under physiological conditions.^108^ OA was traditionally regarded as a non-inflammatory condition, with ST samples from OA patients being used as controls for comparative studies on the pathogenesis of rheumatoid arthritis (RA). Nevertheless, mounting evidence has revealed the presence of inflammation in the ST of individuals with OA.^109,110^

The native synovium appears as a thin layer of tissue, merely one or two cells thick. However, the inflamed synovium in OA experiences cascading changes, including inflammatory edema, fibrosis, and thickening.^111^ Inflammation-related features are observed in the ST of patients with OA including synovial hyperplasia, neovascularization, and infiltration of macrophages and lymphocytes.^112^ While inflammation is crucial for tissue healing, particularly in cases of injury, chronic inflammation has predominantly harmful effects on the joints.^113^ Recent clinical evidence strongly suggests that chronic synovitis accelerates the progression of OA compared to individuals without persistent inflammation.^114^ The innervation of nociceptive sensory nerves in synovium makes it one of the sources of joint pain.^115–119^ MRI studies examining patients with knee OA have established a clear association between synovitis and pain.^120^ This highlights the pivotal role of the synovium as a primary treatment target for mitigating OA-related pain. Despite the strong correlation between synovitis and pain in OA, the precise mechanisms underlying this relationship remain elusive and necessitate further investigation.

In previous studies, the elevated PGE2 level and decreased innervation of sympathetic nerves have been obversed in the inflammatory ST of OA. Therefore, we speculate that the skeletal interoception is a potential mechanism of OA synovitis. In this section, we discuss the potential role of skeletal interoception in OA synovitis.

### Increased PGE2 level is observed in the OA synovium

PGE2, a product derived from arachidonic acid through specific enzymatic cascades, can be triggered by inflammation or injury, as seen in cases of OA.^121,122^ PGE2 is produced through the cyclooxygenase (COX) pathway, with the rate-limiting enzyme COX-2 playing a crucial role in the synthesis of PGE2.^121^ Once the synovium is stimulated by inflammation or injury of the surrounding tissues within the joint, pro-inflammatory mediators such as PGE2 are released by inflammatory macrophages and fibroblasts in the ST of individuals with OA, consequently leading to a marked elevation of PGE2 levels in both the synovium and synovial fluid.^123^ Consequently, the quantification of PGE2 levels in both the synovium and synovial fluid has become a crucial parameter for gauging the effectiveness of drug interventions aimed at addressing synovitis in OA animal models.^124,125^ Pro-inflammatory mediators, such as PGE2, disturb the intricate equilibrium of cartilage metabolism, instigating an excessive release of enzymes that degrade cartilage. Furthermore, fibroblast-like synovial cells engulf deteriorated cartilage particles, intensifying the inflammation of the synovium and forming a vicious cycle.^126,127^

Given the consensus regarding the widespread innervation of sensory nerves in the synovium,^128^ as previously mentioned, we speculate that the elevated levels of PGE2 in the synovium may potentially regulate synovial inflammation through the skeletal interoceptive circuit. To elucidate this matter, it is crucial for future investigations to explore whether the increased PGE2 in the synovium, similar to the PGE2 in subchondral bone, interacts with corresponding receptors (EP1-4) on synovial sensory nerves, thereby transmitting interoceptive signals to the brain.

### The innervation of sympathetic nerves decreased in the synovium of advanced OA

As a bridging interface connecting the brain and the immune system, the SNS participates in modulating synovial inflammation.^129^ Multiple immune cells, including dendritic cells, B lymphocytes, T lymphocytes, macrophages, and natural killer cells, express AR, making them sensitive to NE.^130^ Since the 1950s, the prevailing belief is that sympathetic nerves exacerbate inflammation—for example, in RA.^131,132^ However, a shift in the dominant viewpoint now suggests that the impact of the SNS on ST is contingent upon the specific phase of inflammation.^133^ During the initial acute phase of experimental arthritis, the SNS assumes a pro-inflammatory role, which facilitates the clearance of antigens in the short-term. If the antigens cannot be completely eliminated, it transitions into the chronic inflammatory phase,^130^ in which SNS exhibits intriguing anti-inflammatory effects.^134,135^ Analogous effects of the SNS have also been documented in two models of chronic inflammatory bowel disease.^136^ While the mechanisms facilitating the transition from proinflammatory to anti-inflammatory effects remain enigmatic, we were firmly convinced that these mechanisms contribute to elucidate the dual role of SNS in inflammatory diseases.

Whether the sympathetic nerve sprouting and activity in OA ST are regulated by PGE2-mediated skeletal interoception signals remains unknown. However, the loss of sympathetic nerve fibers in ST during the chronically inflamed stage of RA was reported.^137^ A massive destruction of the neural network was also observed in the OA synovium which is present in normal synovium. Eitner et al. demonstrated that the inflamed OA synovium exhibited a loss of sensory nerve and sympathetic nerve fibers compared to the control synovium.^138^ The above findings are consistent with the conclusion that the SNS exhibits anti-inflammatory effects during the chronic inflammation phase.

**Fig. 4:**
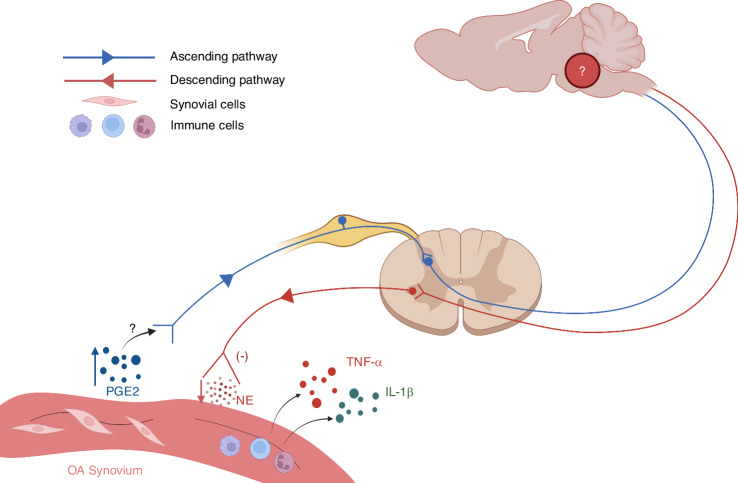
The potential synovial interoception circuit in OA synovitis. The PGE2 level is increased in ST of OA. The elevated PGE2 may activate EP receptors on sensory nerves, thus transmitting the interoceptive signals to the brain (need verification). The brain, after integrating and interpreting interoceptive signals, downregulates sympathetic nerve activity. The SNS exhibits anti-inflammatory effects during the chronic inflammation phase of the synovium, while suppressed sympathetic activity promotes synovial inflammation and the release of pro-inflammatory cytokines such as TNF-α and IL-1β. Further verifying the existence of synovial interoception circuit is warranted, as well as a more comprehensive exploration of the cerebral nucleis involved in synovial interoception. PGE2 prostaglandin E2, ST synovial tissue, EP prostaglandin E, SNS sympathetic nervous system, TNF-α tumor necrosis factor α, IL-1β interleukin 1β, NE norepinephrine

A localized SNS-immune axis has been proven to modulate joint inflammation through vagus nerve stimulation.^139^ The locus coeruleus (LC) and rostral ventrolateral medulla (RVLM), as vital centers of sympathetic regulation, also participate in the anti-inflammatory processes of Parkinson’s disease, fungal toxin-induced inflammatory changes, and joint inflammation.^140,141^ Interestingly, it is widely recognized that activating the brain reward system can stimulate immune system activation, which is inhibited by ablating catecholaminergic neurons comprising the SNS,^142,143^ while stress can also enhance sympathetic activity.^144^ This further confirms the SNS-immune axis, and suggests that the mental state and social environment may participate in the regulation of synovial inflammation by affectting sympathetic activity, although this requires further confirmation. In summary, the relationship between SNS and immunity, as well as inflammation is complex and requires further exploration. This complexity persists in the context of OA and RA as well. Given the decreased innervation of sympathetic nerves in chronic synoviul inflammation, we propose a daring conjecture that the elevated PGE2 in inflamed synovium transmits skeletal interoception signals, or what we may term as “synovium interoception” signals to the brain, which reduces sympathetic nerve activity and potentially further promote synovial inflammation (Fig. 4). By delving into the interoception circuit involved in synovial inflammation and exploring the cerebral nuclei and related mechanisms that modulate this process, we may glean invaluable insights that could inspire breakthroughs in the treatment of synovial inflammation in both OA and RA.

## Targeting skeletal interoception is a promising therapy for OA and its pain

OA often leads to chronic joint pain, physical impairment and even disability, which significantly affects the life quality of patients with OA.^145^ OA affects over 500 million people worldwide and has imposed significant socioeconomic burden due to the aging population.^6^ Unfortunately, no drugs are currently available to reverse OA and clinical analgesic strategies for managing OA pain have been constrained by suboptimal effectiveness and significant adverse effects, which leaves end-stage patients with limited treatment options, such as joint replacement surgery.^146^ Nevertheless, skeletal interoception revealing the role of PGE2 in subchondral bone and bone metabolism offers promising therapeutic prospects for counteracting the progression of OA and mitigating associated pain. In this section, we discuss the critical role of inhibiting PGE2 in subchondral bone, emphasizing its potential for attenuating OA progression and pain. Additionally, we explore the intriguing possibility of activating the SNS to alleviate synovial inflammation and synovium-derived pain in OA.

### Inhibition of PGE2 level suppresses aberrant subchondral bone remodeling and joint pain

Nonsteroidal anti-inflammatory drugs (NSAIDs), such as celecoxib, are first-line medications for treating pain in OA.^147^ Celecoxib primarily alleviates joint pain and inflammation by selectively inhibiting COX-2 activity and reducing PGE2 levels.^148^ However, the exact mechanisms by which PGE2 mediates OA pain have not been fully elucidated. Through the exploration of PGE2 interoception, it has been discovered that inhibiting PGE2 suppresses the progression of OA. This implies that celecoxib holds potential as a disease-modifying drug for OA treatment.

**Fig. 5:**
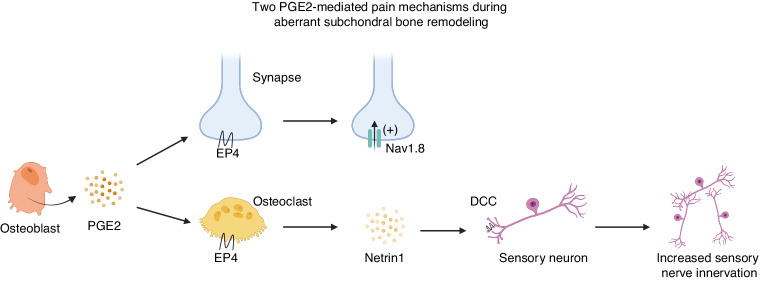
Two PGE2-mediated mechanisms of OA pain. During aberrant subchondral bone remodeling, OBs produced PGE2 binds to EP4 receptors on CGRP^+^ sensory nerves, sensitizing sensitize DRG neurons by modifying the voltage-gated sodium channel Nav1.8. PGE2 also stimulates OCs through EP4 receptors. Activated OCs release netrin1 to activated OCs release netrin1, which binds to the DCC receptors on sensory neurons, promoting the innervation of CGRP^+^ sensory nerves in the subchondral bone. OB osteoblast, PGE2 prostaglandin E2, EP prostaglandin E, CGRP calcitonin-gene-related peptide, DRG dorsal root ganglia, OC osteoclast, DCC deleted in colorectal cancer

PGE2 is involved in two mechanisms contributing to OA pain: nociceptive sensitization and innervation of sensory nerves in subchondral bone (Fig. 5). PGE2 binding to EP1 or EP4 receptors on sensory nerves increases membrane excitability, leading to the activation of nociceptive receptors, which is a classical mechanism underlying PGE2-induced pain.^121^ During abnormal subchondral bone remodeling, PGE2 produced by OBs binds to EP4 receptors on CGRP^+^ sensory nerves, sensitizing sensitize DRG neurons by modifying the voltage-gated sodium channel Nav1.8.^15^ Deletion of the major PGE2 producing enzyme COX-2 in OBs, EP4 receptors in sensory nerves, or inhibition of Nav1.8 in DRG neurons significantly alleviates OA symptoms.^13,15,16^ Therefore, PGE2 and the neuronal modification of Nav1.8 induced by abnormal subchondral bone remodeling are potential therapeutic targets for both OA and other skeletal degenerative disorders.^15^ Investigating how PGE2 produced by synovial pro-inflammatory macrophages and fibroblasts mediates sensory nerve sensitization during the chronic inflammation process in OA is worthwhile.^123^

During the abnormal subchondral bone remodeling, PGE2 produced by OBs activates OCs through the EP4 receptor.^14^ Additionally, abnormal mechanical loading stimulates bone resorption by OCs, leading to the porous structures of subchondaral bone.^149^ Activated OCs produce various factors, among which netrin1 plays a pivotal role in promoting CGRP^+^ nerve sprouting by binding to the DCC receptor on sensory nerves (Fig. 3).^150,151^ Therefore, OCs in the subchondral bone induce sensory nerve innervation and contribute to OA pain. In OA mice model, deletion of the EP4 receptor in OCs or EP4 receptor antagonist significantly alleviates OA pain symptom, accompanied by reduced secretion of netrin1 by OCs and decreased innervation of sensory nerves in subchondral bone.^14^ Moreover, knockout of netrin1 in OCs or the DCC on sensory nerves significantly reduces pain behavior in OA mice.^151^ Importantly, inhibiting OC activity using alendronate in early-stage OA not only significantly suppresses abnormal subchondral bone remodeling but also reduces the innervation of sensory nerves and pain behavior.^151^ These findings highlight another potential mechanism for celecoxib’s inhibition of OA pain and provide a range of targets for treating OA pain.

Considering the classical physiological function of PGE2, the COX-2 inhibitor celecoxib has been widely used in clinical practice as a drug to counter inflammation-related pain. However, in 2014, Doschak et al. developed a multi-modality scoring system for evaluating the progression of OA. Using this research system, they found that in the rat model of post-traumatic OA (PTOA), clinical doses of celecoxib did not exhibit cartilage-protective effects but significantly reduced osteophyte formation compared to a placebo.^152^ The initial mechanism behind this phenomenon was unclear, but the authors speculated that it could be attributed to the upregulation of PGE2-induced receptor activator of NF-κB ligand (RANKL). This, in turn, stimulated bone resorption at the sites of osteophytosis, further contributing to the expansion of osteophyte.^153^ Subsequent studies conducted in 2018 and 2019 revealed that in preclinical OA models, both local intra-articular administration and oral administration of specific doses of celecoxib significantly reduced subchondral bone sclerosis, bone cysts, and osteophyte formation.^16,154^ However, it wasn’t until the successful exploration of skeletal interoception that a reasonable explanation for these findings seemed to emerge. This is because inhibiting PGE2, inducing the sensory denervation, or deleting EP4 on sensory nerves in subchondral bone all significantly attenuate the progression of OA.^13^

It is worth noting that the organism requires a physiological concentration of PGE2 to maintain normal bone mass through the skeletal interoception circuit.^10^ The dosage of celecoxib has an impact on its therapeutic effects. For example, in mice with lumbar spine instability, high doses of celecoxib (80 mg/kg per day) have temporary analgesic effects but severely affect bone mass and lead to increased porosity of the endplate, which provides space for the innervation of sensory nerves. Interestingly, low doses of celecoxib (20 mg/kg per day, one-fourth of the clinical maximum dose) can maintain PGE2 at a physiological concentration, which preserves the bony structure of the endplate with reduced innervation of sensory nerves and alleviated spinal pain. The abirritation persists even after discontinuing treatment.^155^ Therefore, we speculate that low-dose celecoxib can not only inhibit abnormal subchondral bone remodeling and attenuate the progression of OA but also provide persistent inhibition of OA pain. We have designed relevant basic experiments and clinical studies to investigate this further.

### Activation of SNS relieves synovitis and joint pain

It is probably that sympathetic stimuli are effective in combating late-phase joint inflammation, which may be achieved by boosting interleukin-10 production or via the direct anti-inflammatory properties of TH-positive cells.^130^ In late-phase arthritis, sympathetic nerve fibers are pulled away from the site of inflammation and secondary lymphoid organs. Immune cells are generally exposed to low concentrations of NE (<10^−7^ mol/L) released from sympathetic nerve terminals, with α-adrenergic mechanisms predominating.^130^ The fact that sympathectomy results in a pro-inflammatory effect in the late phase of collagen-induced arthritis (CIA) model further suggests that the SNS plays an anti-inflammatory role at this stage.^135^ Therefore, activation of the SNS in the chronic inflammatory phase may have a potential role in resisting synovial inflammation. It has been shown that Prokr2-positive neurons in the ST36 acupoint mediate the activation of SNS during acute inflammation.^156^ Based on ST36 acupoint, Chen and colleagues found that electroacupuncture at ipsilateral ST36-GB34 acupoints in OA rats significantly alleviates synovitis and joint pain by activating the local sympathetic noradrenergic signaling, suggesting a potential therapeutic effect for OA.^157^ Apart from electroacupuncture, sympathectomy in the early inflammatory phase or α-adrenergic agonists may also be potential approaches for treating OA synovitis.

Skeletal interoception not only provides new insights into the pathogenesis and pain mechanisms of OA, but also offers new targets for the treatment of this disease, thereby refreshing our understanding of some first-line clinical drugs. Further exploration of the skeletal interoception mechanisms, including the production of interoceptive signals, ascending sensory nerves, information processing in the brain, and descending SNS, may contribute to the development of novel therapies for OA and its pain.

## Conclusion and perspective

**Fig. 6:**
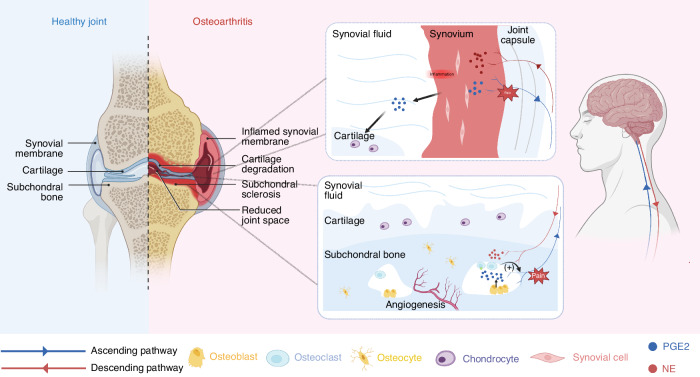
Skeletal interoception in OA and its pain. In advanced OA, there is a progressive deterioration of articular cartilage, along with the development of subchondral bone sclerosis, osteophyte, bone cysts, and synovial inflammation. Elevated PGE2 in subchondral bone transmits interoceptive signals to the brain, which downregulated SNS activity to promote subchondral bone formation and sclerosis. PGE2 also activates OCs to release netrin1, which promotes sensory nerve innervation in the subchondral bone. In the chronic inflammatory phase of OA synovium, PGE2 level is elevated accompanied by decreased sympathetic nerve innervation. This suggests that the skeletal interoception circuit may be involved. The PGE2 in ST can also be released into synovial fluid promoting cartilage destruction. PGE2 prostaglandin E2, SNS sympathetic nervous system, OC osteoclast, ST synovial tissue, NE norepinephrine

OA involves complex pathological processes in which the skeletal interoception circuit regulates subchondral bone remodeling and synovial inflammation (Fig. 6). With the progression of OA, the level of PGE2 in subchondral bone increases, which is usually derived from OBs or osteocytes. The elevated PGE2 can be perceived by EP4 receptors on sensory nerves in subchondral bone and converted into the interoceptive signal. The skeletal interoception signal is transmitted to the brain for integration and interpretation, and current research suggests that the hypothalamus plays a major role in this process. The brain transmits the regulating signals through descending sympathetic nerves to subchondral bone, ultimately promoting subchondral bone sclerosis and the formation of osteophytes. Additionally, the elevated PGE2 in subchondral bone mediates OA pain through two mechanisms: sensitization of sensory neurons and promotion of sensory nerve innervation. Given that previous studies have shown elevated PGE2 level accompanied by reduced sympathetic nerve innervation in ST in the chronic inflammatory phase of OA, it is possible that the skeletal interoception mechanism also participates in the pathogenesis of synovitis in OA, but this requires further validation. The exploration of skeletal interoception provides new therapies for the treatment of OA, such as inhibition of PGE2, antagonism of EP4 receptors, and activation of the SNS. This also challenges the therapeutic mechanisms and dosing of some first-line drugs for OA, such as whether low-dose celecoxib can inhibit the progression of OA and provide sustained relief from joint pain. However, The investigation of skeletal interoception is still in its nascent stages. Whether there are other signal molecules mediating skeletal interoception and which brain regions or nucleis participate in the skeletal interoceptive circuit remains to be further explored. In 2021, the US National Institutes of Health (NIH) released a request for applications “Notice of Special Interest (NOSI): Promoting Research on Interoception and Its Impact on Health and Disease”. It is undeniable that further exploration of skeletal interoception will contribute to our understanding of OA and the development of novel therapies.

## References

[1] Quadt L, Critchley HD, Garfinkel SN (2018). The neurobiology of interoception in health and disease. Ann. N. Y. Acad. Sci..

[2] Shu S (2023). Machine-learning assisted electronic skins capable of proprioception and exteroception in soft robotics. Adv. Mater..

[3] Park HD, Blanke O (2019). Coupling inner and outer body for self-consciousness. Trends Cogn. Sci..

[4] Riddle RC, Clemens TL (2017). Bone cell bioenergetics and skeletal energy homeostasis. Physiol. Rev..

[5] Lv X, Gao F, Cao X (2022). Skeletal interoception in bone homeostasis and pain. Cell Metab..

[6] Yao Q (2023). Osteoarthritis: pathogenic signaling pathways and therapeutic targets. Signal Transduct. Target Ther..

[7] Hunter DJ, Bierma-Zeinstra S (2019). Osteoarthritis. Lancet.

[8] Rizzo MG (2023). Therapeutic perspectives for inflammation and senescence in osteoarthritis using mesenchymal stem cells, mesenchymal stem cell-derived extracellular vesicles and senolytic agents. Cells.

[9] Defois A (2023). Osteoarthritic chondrocytes undergo a glycolysis-related metabolic switch upon exposure to IL-1b or TNF. Cell Commun. Signal.

[10] Chen H (2019). Prostaglandin E2 mediates sensory nerve regulation of bone homeostasis. Nat. Commun..

[11] Collins JA (2023). Cartilage-specific Sirt6 deficiency represses IGF-1 and enhances osteoarthritis severity in mice. Ann. Rheum. Dis..

[12] Zappia J (2023). Osteomodulin downregulation is associated with osteoarthritis development. Bone Res..

[13] Sun Q (2022). Inhibition of PGE2 in subchondral bone attenuates osteoarthritis. Cells.

[14] Jiang W (2022). PGE2 activates EP4 in subchondral bone osteoclasts to regulate osteoarthritis. Bone Res..

[15] Zhu J (2020). Aberrant subchondral osteoblastic metabolism modifies Na(V)1.8 for osteoarthritis. Elife.

[16] Tu M (2019). Inhibition of cyclooxygenase-2 activity in subchondral bone modifies a subtype of osteoarthritis. Bone Res..

[17] Levine DN (2007). Sherrington’s “The integrative action of the nervous system”: a centennial appraisal. J. Neurol. Sci..

[18] Khalsa SS (2018). Interoception and mental health: a roadmap. Biol. Psychiatry Cogn. Neurosci. Neuroimaging.

[19] Chen WG (2021). The emerging science of interoception: sensing, integrating, interpreting, and regulating signals within the self. Trends Neurosci..

[20] Berntson GG, Khalsa SS (2021). Neural circuits of interoception. Trends Neurosci..

[21] Brazill JM, Beeve AT, Craft CS, Ivanusic JJ, Scheller EL (2019). Nerves in bone: evolving concepts in pain and anabolism. J. Bone Min. Res..

[22] Mantyh PW (2014). The neurobiology of skeletal pain. Eur. J. Neurosci..

[23] Holt MK (2019). Synaptic inputs to the mouse dorsal vagal complex and its resident preproglucagon neurons. J. Neurosci..

[24] Lowenstein, E. D. et al. Prox2 and Runx3 vagal sensory neurons regulate esophageal motility. *Neuron* **111**, 2184-2200 (2023). 10.1016/j.neuron.2023.04.025 37192624

[25] Chang RB, Strochlic DE, Williams EK, Umans BD, Liberles SD (2015). Vagal sensory neuron subtypes that differentially control breathing. Cell.

[26] Waise TMZ, Dranse HJ, Lam TKT (2018). The metabolic role of vagal afferent innervation. Nat. Rev. Gastroenterol. Hepatol..

[27] Ly, T. et al. Sequential appetite suppression by oral and visceral feedback to the brainstem. *Nature* **624**, 130-137 (2023). 10.1038/s41586-023-06758-2 PMC10700140 37993711

[28] Critchley HD, Harrison NA (2013). Visceral influences on brain and behavior. Neuron.

[29] Kowalski JL (2023). Resting state functional connectivity differentiation of neuropathic and nociceptive pain in individuals with chronic spinal cord injury. Neuroimage Clin..

[30] Saper CB (2002). The central autonomic nervous system: conscious visceral perception and autonomic pattern generation. Annu. Rev. Neurosci..

[31] Phillips JW (2019). A repeated molecular architecture across thalamic pathways. Nat. Neurosci..

[32] Saper CB, Lowell BB (2014). The hypothalamus. Curr. Biol..

[33] Chiang MC (2019). Parabrachial complex: a hub for pain and aversion. J. Neurosci..

[34] Lathe R, Singadia S, Jordan C, Riedel G (2020). The interoceptive hippocampus: mouse brain endocrine receptor expression highlights a dentate gyrus (DG)-cornu ammonis (CA) challenge-sufficiency axis. PLoS One.

[35] Alexander L (2019). Fractionating blunted reward processing characteristic of anhedonia by over-activating primate subgenual anterior cingulate cortex. Neuron.

[36] Evrard HC (2019). The organization of the primate insular cortex. Front. Neuroanat..

[37] McDougall SJ, Guo H, Andresen MC (2017). Dedicated C-fibre viscerosensory pathways to central nucleus of the amygdala. J. Physiol..

[38] Eickhoff SB (2006). Segregation of visceral and somatosensory afferents: an fMRI and cytoarchitectonic mapping study. Neuroimage.

[39] Joyce MKP, Barbas H (2018). Cortical connections position primate area 25 as a keystone for interoception, emotion, and memory. J. Neurosci..

[40] Namkung H, Kim SH, Sawa A (2017). The insula: an underestimated brain area in clinical neuroscience, psychiatry, and neurology. Trends Neurosci..

[41] Critchley HD, Wiens S, Rotshtein P, Ohman A, Dolan RJ (2004). Neural systems supporting interoceptive awareness. Nat. Neurosci..

[42] Khalsa SS, Rudrauf D, Feinstein JS, Tranel D (2009). The pathways of interoceptive awareness. Nat. Neurosci..

[43] Gu X, Hof PR, Friston KJ, Fan J (2013). Anterior insular cortex and emotional awareness. J. Comp. Neurol..

[44] Abdo H (2019). Specialized cutaneous Schwann cells initiate pain sensation. Science.

[45] Hanoun M, Maryanovich M, Arnal-Estape A, Frenette PS (2015). Neural regulation of hematopoiesis, inflammation, and cancer. Neuron.

[46] Elefteriou F (2018). Impact of the autonomic nervous system on the skeleton. Physiol. Rev..

[47] Kobori N, Moore AN, Redell JB, Dash PK (2023). Caudal DMN neurons innervate the spleen and release CART peptide to regulate neuroimmune function. J. Neuroinflammation.

[48] Takano T, Yule DI (2023). Ca^2+^ signals in pancreatic acinar cells in response to physiological stimulation in vivo. J. Physiol..

[49] Askmyr M, Quach J, Purton LE (2011). Effects of the bone marrow microenvironment on hematopoietic malignancy. Bone.

[50] Zhou R (2021). Endocrine role of bone in the regulation of energy metabolism. Bone Res..

[51] Bu T, Zheng J, Liu L, Li S, Wu J (2021). Milk proteins and their derived peptides on bone health: biological functions, mechanisms, and prospects. Compr. Rev. Food Sci. Food Saf..

[52] Crane JL, Cao X (2014). Bone marrow mesenchymal stem cells and TGF-beta signaling in bone remodeling. J. Clin. Invest..

[53] Hu B (2022). Sensory nerve maintains intervertebral disc extracellular matrix homeostasis via CGRP/CHSY1 axis. Adv. Sci. (Weinh.).

[54] Xiao Y (2023). Interoceptive regulation of skeletal tissue homeostasis and repair. Bone Res..

[55] Goldring MB, Goldring SR (2010). Articular cartilage and subchondral bone in the pathogenesis of osteoarthritis. Ann. N. Y. Acad. Sci..

[56] Holopainen JT (2008). Changes in subchondral bone mineral density and collagen matrix organization in growing horses. Bone.

[57] Hu W (2020). Tumour dormancy in inflammatory microenvironment: a promising therapeutic strategy for cancer-related bone metastasis. Cell Mol. Life Sci..

[58] Hu W, Chen Y, Dou C, Dong S (2021). Microenvironment in subchondral bone: predominant regulator for the treatment of osteoarthritis. Ann. Rheum. Dis..

[59] Hu Y, Chen X, Wang S, Jing Y, Su J (2021). Subchondral bone microenvironment in osteoarthritis and pain. Bone Res..

[60] Hugle T, Geurts J (2017). What drives osteoarthritis?-synovial versus subchondral bone pathology. Rheumatology (Oxford).

[61] Zhen G (2021). Mechanical stress determines the configuration of TGFbeta activation in articular cartilage. Nat. Commun..

[62] Burr DB (2004). Anatomy and physiology of the mineralized tissues: role in the pathogenesis of osteoarthrosis. Osteoarthr. Cartil..

[63] Chen Y (2017). Abnormal subchondral bone remodeling and its association with articular cartilage degradation in knees of type 2 diabetes patients. Bone Res..

[64] Suri S (2007). Neurovascular invasion at the osteochondral junction and in osteophytes in osteoarthritis. Ann. Rheum. Dis..

[65] Tomlinson RE (2017). NGF-TrkA signaling in sensory nerves is required for skeletal adaptation to mechanical loads in mice. Proc. Natl. Acad. Sci. USA.

[66] Xie H (2014). PDGF-BB secreted by preosteoclasts induces angiogenesis during coupling with osteogenesis. Nat. Med..

[67] Gao X (2023). Leptin receptor(+) cells promote bone marrow innervation and regeneration by synthesizing nerve growth factor. Nat. Cell Biol..

[68] Hatch RJ, Jennings EA, Ivanusic JJ (2013). Peripheral hyperpolarization-activated cyclic nucleotide-gated channels contribute to inflammation-induced hypersensitivity of the rat temporomandibular joint. Eur. J. Pain..

[69] Nagae M, Hiraga T, Yoneda T (2007). Acidic microenvironment created by osteoclasts causes bone pain associated with tumor colonization. J. Bone Min. Metab..

[70] Kao FC, Chiu PY, Tsai TT, Lin ZH (2019). The application of nanogenerators and piezoelectricity in osteogenesis. Sci. Technol. Adv. Mater..

[71] Robling AG, Castillo AB, Turner CH (2006). Biomechanical and molecular regulation of bone remodeling. Annu. Rev. Biomed. Eng..

[72] Elmaleh-Sachs A (2023). Obesity management in adults: a review. JAMA.

[73] Wheeler, T. A. et al. Mechanical loading of joint modulates T cells in lymph nodes to regulate osteoarthritis. *Osteoarthritis Cartilage* (2023). 10.1016/j.joca.2023.11.021 PMC10955501 38072172

[74] Jin Y (2022). Carbon dots derived from folic acid attenuates osteoarthritis by protecting chondrocytes through NF-kappaB/MAPK pathway and reprogramming macrophages. J. Nanobiotechnol..

[75] Prinz, E. et al. OA susceptibility in mice is partially mediated by the gut microbiome, is transferrable via microbiome transplantation and is associated with immunophenotype changes. *Ann. Rheum. Dis.* **83**, 382-393 (2023). 10.1136/ard-2023-224907 PMC10922159 37979958

[76] Ding R, Zhang N, Wang Q, Wang W (2022). Alterations of the subchondral bone in osteoarthritis: complying with Wolff’s Law. Curr. Rheumatol. Rev..

[77] Han X (2022). Abnormal subchondral trabecular bone remodeling in knee osteoarthritis under the influence of knee alignment. Osteoarthr. Cartil..

[78] Waterson MJ, Horvath TL (2015). Neuronal regulation of energy homeostasis: beyond the hypothalamus and feeding. Cell Metab..

[79] Blackwell KA, Raisz LG, Pilbeam CC (2010). Prostaglandins in bone: bad cop, good cop?. Trends Endocrinol. Metab..

[80] Guo, Q. et al. Unloading-induced skeletal interoception alters hypothalamic signaling to promote bone loss and fat metabolism. *Adv. Sci. (Weinh)* **10**, e2305042 (2023). 10.1002/advs.202305042 PMC10724445 37880864

[81] Bailey KN (2021). Mechanosensitive control of articular cartilage and subchondral bone homeostasis in mice requires osteocytic transforming growth factor beta signaling. Arthritis Rheumatol..

[82] Sanchez C, Gabay O, Salvat C, Henrotin YE, Berenbaum F (2009). Mechanical loading highly increases IL-6 production and decreases OPG expression by osteoblasts. Osteoarthr. Cartil..

[83] Goldring SR (2009). Role of bone in osteoarthritis pathogenesis. Med. Clin. North Am..

[84] Yoshida K (2002). Stimulation of bone formation and prevention of bone loss by prostaglandin E EP4 receptor activation. Proc. Natl. Acad. Sci. USA.

[85] Chartier SR, Mitchell SAT, Majuta LA, Mantyh PW (2018). The changing sensory and sympathetic innervation of the young, adult and aging mouse femur. Neuroscience.

[86] Gil, C. M. et al. Myostatin and CXCL11 promote nervous tissue macrophages to maintain osteoarthritis pain. *Brain Behav. Immun.* **116**, 203-215 (2023). 10.1016/j.bbi.2023.12.004 38070625

[87] Tomlinson RE (2016). NGF-TrkA signaling by sensory nerves coordinates the vascularization and ossification of developing endochondral bone. Cell Rep..

[88] Fong H, Zheng J, Kurrasch D (2023). The structural and functional complexity of the integrative hypothalamus. Science.

[89] Yang F (2020). A GABAergic neural circuit in the ventromedial hypothalamus mediates chronic stress-induced bone loss. J. Clin. Invest..

[90] Oury F (2010). CREB mediates brain serotonin regulation of bone mass through its expression in ventromedial hypothalamic neurons. Genes Dev..

[91] Lv X (2021). Skeleton interoception regulates bone and fat metabolism through hypothalamic neuroendocrine NPY. Elife.

[92] Takeda S (2002). Leptin regulates bone formation via the sympathetic nervous system. Cell.

[93] Guo Q (2023). Sympathetic innervation regulates osteocyte-mediated cortical bone resorption during lactation. Adv. Sci. (Weinh.).

[94] Elefteriou F (2005). Leptin regulation of bone resorption by the sympathetic nervous system and CART. Nature.

[95] Zhang Y (2021). Neuronal induction of bone-fat imbalance through osteocyte neuropeptide Y. Adv. Sci. (Weinh.).

[96] Rosch G (2021). beta2-adrenoceptor deficiency results in increased calcified cartilage thickness and subchondral bone remodeling in murine experimental osteoarthritis. Front. Immunol..

[97] Rosch G (2022). Sympathectomy aggravates subchondral bone changes during osteoarthritis progression in mice without affecting cartilage degeneration or synovial inflammation. Osteoarthr. Cartil..

[98] Rosch G, Zaucke F, Jenei-Lanzl Z (2023). Autonomic nervous regulation of cellular processes during subchondral bone remodeling in osteoarthritis. Am. J. Physiol. Cell Physiol..

[99] Hu B (2020). Sensory nerves regulate mesenchymal stromal cell lineage commitment by tuning sympathetic tones. J. Clin. Invest..

[100] Loh K, Herzog H, Shi YC (2015). Regulation of energy homeostasis by the NPY system. Trends Endocrinol. Metab..

[101] Garcia TB, Hollborn M, Bringmann A (2017). Expression and signaling of NGF in the healthy and injured retina. Cytokine Growth Factor Rev..

[102] Jimenez-Andrade JM (2010). A phenotypically restricted set of primary afferent nerve fibers innervate the bone versus skin: therapeutic opportunity for treating skeletal pain. Bone.

[103] Castaneda-Corral G (2011). The majority of myelinated and unmyelinated sensory nerve fibers that innervate bone express the tropomyosin receptor kinase A. Neuroscience.

[104] Li Z (2019). Fracture repair requires TrkA signaling by skeletal sensory nerves. J. Clin. Invest..

[105] Martel-Pelletier J (2016). Osteoarthritis. Nat. Rev. Dis. Prim..

[106] Rockel JS, Kapoor M (2018). The metabolome and osteoarthritis: possible contributions to symptoms and pathology. Metabolites.

[107] Pandey, A. & Bhutani, N. Profiling joint tissues at single-cell resolution: advances and insights. *Nat. Rev. Rheumatol*. **20**, 7-20 (2023). 10.1038/s41584-023-01052-x PMC11674069 38057475

[108] de Lange-Brokaar BJ (2012). Synovial inflammation, immune cells and their cytokines in osteoarthritis: a review. Osteoarthr. Cartil..

[109] Myers SL (1990). Synovial inflammation in patients with early osteoarthritis of the knee. J. Rheumatol..

[110] Loeuille D (2005). Macroscopic and microscopic features of synovial membrane inflammation in the osteoarthritic knee: correlating magnetic resonance imaging findings with disease severity. Arthritis Rheum..

[111] Knights AJ, Redding SJ, Maerz T (2023). Inflammation in osteoarthritis: the latest progress and ongoing challenges. Curr. Opin. Rheumatol..

[112] Scanzello CR, Goldring SR (2012). The role of synovitis in osteoarthritis pathogenesis. Bone.

[113] Robinson WH (2016). Low-grade inflammation as a key mediator of the pathogenesis of osteoarthritis. Nat. Rev. Rheumatol..

[114] Ramezanpour S (2023). Impact of sustained synovitis on knee joint structural degeneration: 4-year MRI data from the osteoarthritis initiative. J. Magn. Reson Imaging.

[115] Kc R (2016). PKCdelta null mutations in a mouse model of osteoarthritis alter osteoarthritic pain independently of joint pathology by augmenting NGF/TrkA-induced axonal outgrowth. Ann. Rheum. Dis..

[116] Blum R, Kafitz KW, Konnerth A (2002). Neurotrophin-evoked depolarization requires the sodium channel Na(V)1.9. Nature.

[117] Kafitz KW, Rose CR, Konnerth A (2000). Neurotrophin-evoked rapid excitation of central neurons. Prog. Brain Res..

[118] Kafitz KW, Rose CR, Thoenen H, Konnerth A (1999). Neurotrophin-evoked rapid excitation through TrkB receptors. Nature.

[119] InSug O-S (2022). Sensory neuron-specific deletion of tropomyosin receptor kinase A (TrkA) in mice abolishes osteoarthritis (OA) pain via NGF/TrkA intervention of peripheral sensitization. Int. J. Mol. Sci..

[120] Perry TA, Yang X, van Santen J, Arden NK, Kluzek S (2021). Quantitative and semi-quantitative assessment of synovitis on MRI and the relationship with symptoms in symptomatic knee osteoarthritis. Rheumatology (Oxf.).

[121] Martel-Pelletier J, Pelletier JP, Fahmi H (2003). Cyclooxygenase-2 and prostaglandins in articular tissues. Semin. Arthritis Rheum..

[122] Wittenberg RH, Willburger RE, Kleemeyer KS, Peskar BA (1993). In vitro release of prostaglandins and leukotrienes from synovial tissue, cartilage, and bone in degenerative joint diseases. Arthritis Rheum..

[123] Benito MJ, Veale DJ, FitzGerald O, van den Berg WB, Bresnihan B (2005). Synovial tissue inflammation in early and late osteoarthritis. Ann. Rheum. Dis..

[124] Chen Q (2015). Xanthan gum protects rabbit articular chondrocytes against sodium nitroprusside-induced apoptosis in vitro. Carbohydr. Polym..

[125] Hsueh MF, Bolognesi MP, Wellman SS, Kraus VB (2020). Anti-inflammatory effects of naproxen sodium on human osteoarthritis synovial fluid immune cells. Osteoarthr. Cartil..

[126] Sellam J, Berenbaum F (2010). The role of synovitis in pathophysiology and clinical symptoms of osteoarthritis. Nat. Rev. Rheumatol..

[127] Silverstein AM (2017). Toward understanding the role of cartilage particulates in synovial inflammation. Osteoarthr. Cartil..

[128] Kidd BL (1989). A neurogenic mechanism for symmetrical arthritis. Lancet.

[129] Elenkov IJ, Wilder RL, Chrousos GP, Vizi ES (2000). The sympathetic nerve–an integrative interface between two supersystems: the brain and the immune system. Pharm. Rev..

[130] Pongratz G, Straub RH (2013). Role of peripheral nerve fibres in acute and chronic inflammation in arthritis. Nat. Rev. Rheumatol..

[131] Fellinger K, Schmid J, Leonhartsberger F, Hofmann G, Ferstl A (1952). Sympathetic block in primary chronic polyarthritis. Munch. Med. Wochenschr..

[132] Levine JD, Goetzl EJ, Basbaum AI (1987). Contribution of the nervous system to the pathophysiology of rheumatoid arthritis and other polyarthritides. Rheum. Dis. Clin. North Am..

[133] Capellino S (2010). Catecholamine-producing cells in the synovial tissue during arthritis: modulation of sympathetic neurotransmitters as new therapeutic target. Ann. Rheum. Dis..

[134] Ebbinghaus M, Gajda M, Boettger MK, Schaible HG, Brauer R (2012). The anti-inflammatory effects of sympathectomy in murine antigen-induced arthritis are associated with a reduction of Th1 and Th17 responses. Ann. Rheum. Dis..

[135] Harle P, Mobius D, Carr DJ, Scholmerich J, Straub RH (2005). An opposing time-dependent immune-modulating effect of the sympathetic nervous system conferred by altering the cytokine profile in the local lymph nodes and spleen of mice with type II collagen-induced arthritis. Arthritis Rheum..

[136] Straub RH (2008). Anti-inflammatory role of sympathetic nerves in chronic intestinal inflammation. Gut.

[137] Miller LE, Justen HP, Scholmerich J, Straub RH (2000). The loss of sympathetic nerve fibers in the synovial tissue of patients with rheumatoid arthritis is accompanied by increased norepinephrine release from synovial macrophages. FASEB J..

[138] Eitner A, Pester J, Nietzsche S, Hofmann GO, Schaible HG (2013). The innervation of synovium of human osteoarthritic joints in comparison with normal rat and sheep synovium. Osteoarthr. Cartil..

[139] Bassi GS (2017). Modulation of experimental arthritis by vagal sensory and central brain stimulation. Brain Behav. Immun..

[140] Yoon SY (2007). Peripheral bee venom’s anti-inflammatory effect involves activation of the coeruleospinal pathway and sympathetic preganglionic neurons. Neurosci. Res..

[141] O’Neill E, Harkin A (2018). Targeting the noradrenergic system for anti-inflammatory and neuroprotective effects: implications for Parkinson’s disease. Neural Regen. Res..

[142] Ben-Shaanan TL (2016). Activation of the reward system boosts innate and adaptive immunity. Nat. Med..

[143] Ben-Shaanan TL (2018). Modulation of anti-tumor immunity by the brain’s reward system. Nat. Commun..

[144] Zhang B (2020). Hyperactivation of sympathetic nerves drives depletion of melanocyte stem cells. Nature.

[145] Fang H, Beier F (2014). Mouse models of osteoarthritis: modelling risk factors and assessing outcomes. Nat. Rev. Rheumatol..

[146] Mueller AJ, Peffers MJ, Proctor CJ, Clegg PD (2017). Systems approaches in osteoarthritis: identifying routes to novel diagnostic and therapeutic strategies. J. Orthop. Res..

[147] Krasselt M, Baerwald C (2019). Celecoxib for the treatment of musculoskeletal arthritis. Expert Opin. Pharmacother..

[148] Iyer JP, Srivastava PK, Dev R, Dastidar SG, Ray A (2009). Prostaglandin E2 synthase inhibition as a therapeutic target. Expert Opin. Ther. Targets.

[149] Bian Q (2016). Excessive activation of TGFbeta by spinal instability causes vertebral endplate sclerosis. Sci. Rep..

[150] Ni S (2019). Sensory innervation in porous endplates by Netrin-1 from osteoclasts mediates PGE2-induced spinal hypersensitivity in mice. Nat. Commun..

[151] Zhu S (2019). Subchondral bone osteoclasts induce sensory innervation and osteoarthritis pain. J. Clin. Invest..

[152] Panahifar A (2014). Development and reliability of a multi-modality scoring system for evaluation of disease progression in pre-clinical models of osteoarthritis: celecoxib may possess disease-modifying properties. Osteoarthr. Cartil..

[153] Akatsu T (1989). Prostaglandins promote osteoclastlike cell formation by a mechanism involving cyclic adenosine 3’,5’-monophosphate in mouse bone marrow cell cultures. J. Bone Min. Res..

[154] Tellegen AR (2018). Controlled release of celecoxib inhibits inflammation, bone cysts and osteophyte formation in a preclinical model of osteoarthritis. Drug Deliv..

[155] Xue P (2021). PGE2/EP4 skeleton interoception activity reduces vertebral endplate porosity and spinal pain with low-dose celecoxib. Bone Res..

[156] Liu S (2021). A neuroanatomical basis for electroacupuncture to drive the vagal-adrenal axis. Nature.

[157] Chen W (2023). Electroacupuncture activated local sympathetic noradrenergic signaling to relieve synovitis and referred pain behaviors in knee osteoarthritis rats. Front. Mol. Neurosci..

[158] Inoue H (2001). Production of neuropeptide substance P by synovial fibroblasts from patients with rheumatoid arthritis and osteoarthritis. Neurosci. Lett..

[159] Liu L, Dana R, Yin J (2020). Sensory neurons directly promote angiogenesis in response to inflammation via substance P signaling. FASEB J..

[160] Heikkila HM, Hielm-Bjorkman AK, Innes JF, Laitinen-Vapaavuori OM (2017). The effect of intra-articular botulinum toxin A on substance P, prostaglandin E(2), and tumor necrosis factor alpha in the canine osteoarthritic joint. BMC Vet. Res..

[161] Im HJ (2008). Basic fibroblast growth factor accelerates matrix degradation via a neuro-endocrine pathway in human adult articular chondrocytes. J. Cell Physiol..

[162] Warner SC (2017). Pain in knee osteoarthritis is associated with variation in the neurokinin 1/substance P receptor (TACR1) gene. Eur. J. Pain..

[163] Jin Y (2022). A novel prostaglandin E receptor 4 (EP4) small molecule antagonist induces articular cartilage regeneration. Cell Discov..

[164] Walsh DA (2010). Angiogenesis and nerve growth factor at the osteochondral junction in rheumatoid arthritis and osteoarthritis. Rheumatology (Oxf.).

[165] Pecchi E (2014). Induction of nerve growth factor expression and release by mechanical and inflammatory stimuli in chondrocytes: possible involvement in osteoarthritis pain. Arthritis Res. Ther..

[166] Nencini S (2017). Mechanisms of nerve growth factor signaling in bone nociceptors and in an animal model of inflammatory bone pain. Mol. Pain..

[167] Zhen G (2013). Inhibition of TGF-beta signaling in mesenchymal stem cells of subchondral bone attenuates osteoarthritis. Nat. Med..

[168] Stockl S (2021). Substance P and alpha-calcitonin gene-related peptide differentially affect human osteoarthritic and healthy chondrocytes. Front. Immunol..

[169] Bohm M (2016). alpha-MSH modulates cell adhesion and inflammatory responses of synovial fibroblasts from osteoarthritis patients. Biochem. Pharm..

[170] Can VC (2020). Novel anti-inflammatory and chondroprotective effects of the human melanocortin MC1 receptor agonist BMS-470539 dihydrochloride and human melanocortin MC3 receptor agonist PG-990 on lipopolysaccharide activated chondrocytes. Eur. J. Pharm..

[171] Su W (2020). Angiogenesis stimulated by elevated PDGF-BB in subchondral bone contributes to osteoarthritis development. JCI Insight.

[172] Cui Z (2022). Endothelial PDGF-BB/PDGFR-beta signaling promotes osteoarthritis by enhancing angiogenesis-dependent abnormal subchondral bone formation. Bone Res..

[173] Opolka A, Straub RH, Pasoldt A, Grifka J, Grassel S (2012). Substance P and norepinephrine modulate murine chondrocyte proliferation and apoptosis. Arthritis Rheum..

[174] Lorenz J (2016). Norepinephrine modulates osteoarthritic chondrocyte metabolism and inflammatory responses. Osteoarthr. Cartil..

[175] Hwang HS, Lee MH, Go DJ, Kim HA (2021). Norepinephrine modulates IL-1beta-induced catabolic response of human chondrocytes. BMC Musculoskelet. Disord..

[176] El Bagdadi K, Zaucke F, Meurer A, Straub RH, Jenei-Lanzl Z (2019). Norepinephrine inhibits synovial adipose stem cell chondrogenesis via alpha2a-adrenoceptor-mediated ERK1/2 activation. Int. J. Mol. Sci..

[177] Perez-Garcia S (2021). Proteomic analysis of synovial fibroblasts and articular chondrocytes co-cultures reveals valuable VIP-modulated inflammatory and degradative proteins in osteoarthritis. Int. J. Mol. Sci..

[178] Liang Y (2018). Vasoactive intestinal peptide alleviates osteoarthritis effectively via inhibiting NF-kappaB signaling pathway. J. Biomed. Sci..

[179] Perez-Garcia S (2016). VIP and CRF reduce ADAMTS expression and function in osteoarthritis synovial fibroblasts. J. Cell Mol. Med..

[180] Wang L (2014). Levels of neuropeptide Y in synovial fluid relate to pain in patients with knee osteoarthritis. BMC Musculoskelet. Disord..

[181] Ferreira-Gomes J, Adaes S, Sousa RM, Mendonca M, Castro-Lopes JM (2012). Dose-dependent expression of neuronal injury markers during experimental osteoarthritis induced by monoiodoacetate in the rat. Mol. Pain..

[182] Kang X (2020). Neuropeptide Y acts directly on cartilage homeostasis and exacerbates progression of osteoarthritis through NPY2R. J. Bone Min. Res..

[183] Hori A, Nishida T, Takashiba S, Kubota S, Takigawa M (2017). Regulatory mechanism of CCN2 production by serotonin (5-HT) via 5-HT2A and 5-HT2B receptors in chondrocytes. PLoS One.

[184] Stratz C, Anakwue J, Bhatia H, Pitz S, Fiebich BL (2014). Anti-inflammatory effects of 5-HT3 receptor antagonists in interleukin-1beta stimulated primary human chondrocytes. Int. Immunopharmacol..

